# Detection Rate of ^18^F-Fluorethylcholine-PET/CT in relation to PSA Value in PCA Patients Referred with Biochemical Relapse

**DOI:** 10.1155/2020/4320178

**Published:** 2020-08-11

**Authors:** Mustafa Takesh, Khaldoun Odat Allh, Christian M. Zechmann

**Affiliations:** ^1^Department of Nuclear Medicine, Heidelberg University Hospital, Im Neuenheimer Feld 400, 69120 Heidelberg, Germany; ^2^Department of Urology, Heidelberg University Hospital, Im Neuenheimer Feld 110, 69120 Heidelberg, Germany

## Abstract

Attempts to predict the likelihood of positive morphological imaging related with PSA value in patients referred with biochemical recurrence were the focus of many studies. Using nuclear medicine modalities, numerous studies likewise had been performed for the same purpose, however mostly using C-11-labeled choline. For this purpose, we selected 193 prostate cancer patients from our database between 2006 and 2010. They had been referred to our department to undergo ^18^F-fluorethylcholine (FECH)-PET/CT due to biochemical recurrence after potentially curative procedures. As a result, in 84 out of 193 patients, ^18^F-FECH-PET demonstrated positive findings with an overall detection rate of 44%. Statistically, there was a significant difference in PSA values in positive findings vs. negative findings (*p* < 0.001), and there was a linear correlation between the detection rate and PSA value (*r* = 0.91). Moreover, there was a relation between initial therapy and recurrence type. So, the local relapse was the most frequent recurrence (>70%) after radiation therapy alone. By contrast, patients after radical prostatectomy followed by salvage radiotherapy showed a low likelihood of local recurrence. In conclusion, PSA value was confirmed to have a determinant role in ^18^F-FECH-PET outcome. Moreover, there was a link between recurrence type and initial therapy, which—if prospectively confirmed—may play a guiding role in selecting the appropriate diagnostic methods.

## 1. Introduction

It is known that prostate cancer (PCA) is the second most common cause of cancer death in men over 50 years. After a curative procedure, a standard follow-up plan using regular PSA tests, is essential for early detection of recurrence to minimize the morbidity rate.

A rise in PSA is often the single early indicator for recurrence. Therefore, the main point of concern using the radiological or nuclear medicine procedures in patients referred with biochemical failure is about their efficiency in detecting recurrent cancer at low PSA levels.

 While conventional imaging (CI) has a poor accuracy, in particular with moderately elevated prostate-specific antigen (PSA) levels [1–3], PET with radiolabeled choline had been introduced as a high-quality method in both primary and recurrent PCA. The increased choline uptake in prostate cancer cells is thought to be caused by an increased cell proliferation in tumors, as choline is a precursor for the biosynthesis of phosphatidylcholine (the major component of the cell membrane). The mechanism behind that increase is an enhanced activity of choline transport and choline phosphorylation in cancer cells, which was experimentally confirmed in human-derived prostate cancer [4–7]. However, choline-PET still faces the problem of decreased sensitivity at low PSA levels [8, 9]. Moreover, the choline retention is not specific for PCA and may accumulate in many other disorders not related to PCA such as brain and intrathoracic tumors [10–12].

Attempts to predict the likelihood of positive conventional imaging related with PSA value were the focus of many studies. Choueiri et al. [13] used several predictive factors in addition to PSA value, such as Gleason score, primary therapy, and PSA doubling time. Using the nuclear medicine modalities, numerous studies had been performed regarding the diagnostic capability of choline PET in recurrent diseases.

To know how the sensitivity of ^18^F-FECH-PET/CT varies with PSA value and to reveal other factors possible to affect the test sensitivity were the main issues we focused on in this retrospective study. Moreover, we aimed to estimate to which extent the PSA value differs in positive findings versus that in negative findings.

## 2. Materials and Methods

### 2.1. Patients

**Figure 1:**
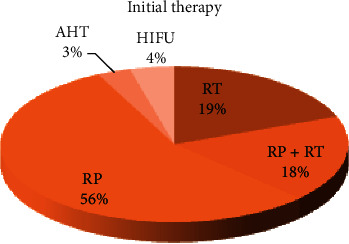
Illustrating the different types of initial therapies. RP, radical prostatectomy; RT, radiotherapy; AHT, antihormonal therapy; HIFU, high-intensity focused ultrasound.

Our database from 2006 to 2010 revealed 193 prostate cancer patients (mean age 66.4 years). The Gleason score of these patients ranged between 4 and 9. They had been referred to our department to undergo ^18^F-FECH-PET/CT due to biochemical recurrence after potentially curative procedure. As demonstrated in Figure 1, the patients had a wide variety of initial therapies, such as radical prostatectomy (55%) or external radiation (19%), and 34 patients (18%) underwent prostatectomy as the primary procedure followed by external radiation due to slight PSA relapse. Six patients developing a PSA increase in spite of ongoing antihormonal therapy were also included. Since the doubling time was not available in all patients, it could not be evaluated as an affecting factor.

### 2.2. Synthesis of ^18^F-FECH

^18^F-FECH was prepared in a two-step synthesis; we first synthesized nonradioactive FECH and then used this compound for synthesis of radioactive (no carrier added) ^18^F-FECH using the TBA method described by Hara et al. [14].

### 2.3. Imaging Protocol

**Figure 2:**
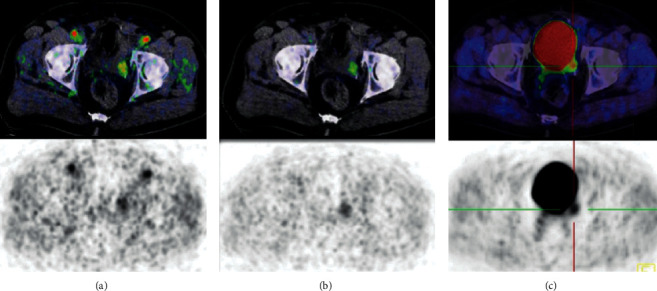
A 70-year-old patient after radical prostatectomy referred with biochemical relapse due to a recurrence in the left seminal vesicle. Transaxial planes in both dynamic and static acquisitions illustrating the benefit of the dynamic phase in detecting the local recurrence. (a) Start of the dynamic phase showing simultaneous appearance of recurrence and arteries (see the radiopharmacon in the arteries). (b) Frame at the end of the dynamic phase. (c) Static imaging showing a focal increased uptake at the posterior wall of bladder, indistinguishable from bladder diverticulum or retained activity in the distal ureter, without considering the dynamic study.

All PET studies were carried out using a Biograph 6 PET/CT (Siemens/CTI). Imaging of the pelvic region was started with a dynamic phase simultaneous to the administration of 250–350 MBq of ^18^F-fluorethylcholine for 10 min in the list mode before proceeding to whole-body imaging at 1 h p.i. The benefit of dynamic study is emphasized if local recurrence is suspected. Thereby, it was possible to assess the prostate bed before arrival of the activity in the bladder (Figure 2).

For attenuation correction of the PET scan, a low-dose CT (130 keV, 30 mAs; care dose) without a contrast medium was performed. Static emission scans, corrected for dead time, scatter, and decay, were acquired from the vertex to the proximal legs, requiring eight bed positions, 4 minutes each. The images were iteratively reconstructed with the OSEM algorithm using four iterations with eight subsets and Gauss filtering to an in-plane spatial resolution of 5 mm at full-width half-maximum.

### 2.4. Image Analysis and Finding Validation

**Figure 3:**
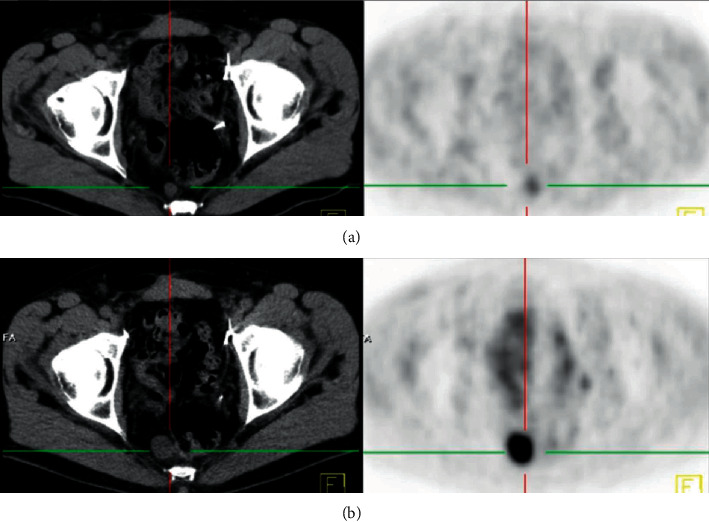
A 64-year-old PCA patient referred with PSA rising (11.5) after radical prostatectomy. The initial ^18^F-fluorethylcholine PET-CT demonstrated mildly increased uptake in a slightly enlarged perirectal lymph node. Six months later, the repeated ^18^F-fluorethylcholine PET-CT exhibited marked increase in both volume and intensity. This interval progression would verify the diagnosis of lymph node metastasis as a cause of PSA relapse.

The images were revised in several displays (transaxial, sagittal, and coronal) and evaluated also considering clinical data in a session of two experienced nuclear medicine physicians and a radiologist. The evaluation was based on visual recognition of focal accumulation of choline higher than the surrounding background level. The dynamic phase was reanalyzed, mainly concerning the status of the prostate bed. Owing to the absence of histological confirmation, the validation of positive findings necessitated a typical PET-finding corresponding with accompanied CT if clinically required with contrast enhancement and/or other radiological modalities, e.g., MRI. Moreover, progression detected in the subsequent follow-up scans was considered as an indicator of malignancy (Figure 3).

### 2.5. Statistical Analysis

The comparison between data had been performed using static graphic program software package on a personal computer (Pentium (R) Dual-Core CPU (2.00 GHz, 3.00 GB random access memory)) running with Windows Vista Home Premium. Descriptive statistics and box plots were used for the analysis of the data. *p* values <0.05 were considered statistically significant.

## 3. Results

### 3.1. Overall Detection Rate

**Figure 4:**
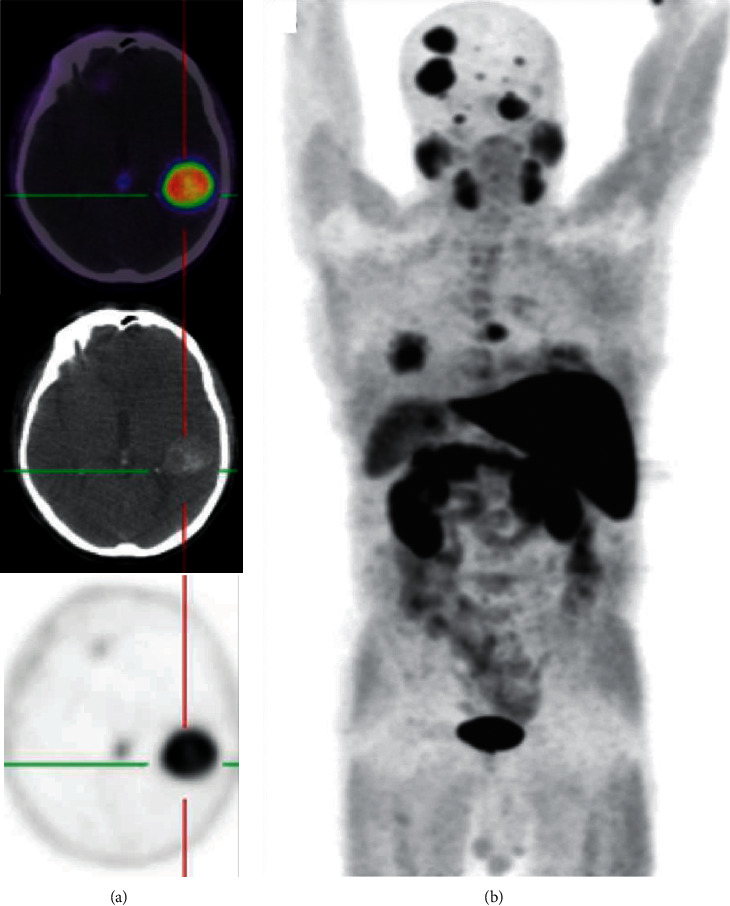
A 61-year-old patient with hormone refractory PCA (initial pT3c pN0 M0) and treated with radical prostatectomy followed by radiation therapy was referred with PSA relapse (PSA serum level: 43 ng/dl). (a) ^18^F-FECH-PET/CT of the brain, axial fused planes (upper image), axial CT plane (middle image), and PET (lower image) showed a delineated increased uptake within the brain parenchyma located in the left temporal lobe matching to a well-circumscribed hyperdense area in CT in terms of a brain metastasis. This was histologically confirmed. Note the high tumor-to-background signal. (b) ^18^F-FECH-PET (MIP) shows numerous supratentorial and infratentorial brain metastases in addition to the lung metastases (posterior view).

In 84 out of 193 patients, ^18^F-FECH-PET demonstrated a positive finding in terms of local recurrence, lymph node recurrence, or bone metastasis with an overall detection rate of 44% (lymph node 27%, local recurrence 11%, and bone metastases 6%.). Unusual recurrence sites were found in two cases, including brain metastasis (Figure 4) and skull base metastasis.

On the other hand, there was a positive FECH uptake in 3 cases not related to PCA in keeping with false-positive findings (meningioma, lung malignancy, and subclavian venous aneurysm).

### 3.2. Detection Rate Linked with PSA

**Figure 5:**
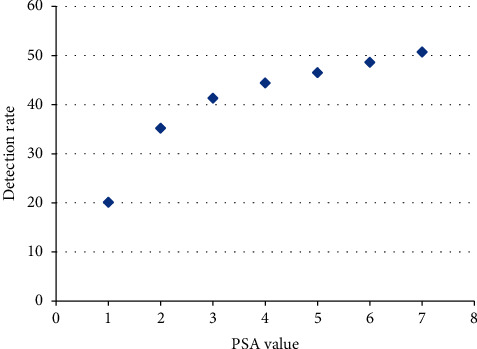
Correlation between detection rate and PSA value (*r* = 0. 91).

The patients were referred with a broad range of PSA values, which is in turn supposed to affect the sensitivity of the ^18^F-FECH-PET. Accordingly, the detection rate (DR) was reviewed with regard to PSA value. In PSA value less than 1 ng/ml, DR was 20%, whereas in case of PSA <1.5 ng/ml, DR was 32%. Further results show a steady increase of DR with PSA as follows: 35%, 41%, 44%, 46%, 48.4%, and 50% in PSA value less than 2 ng/ml, 3 ng/ml, 4 ng/ml, 5 ng/ml, 6 ng/ml, and 7 ng/ml, respectively. As a consequence, there was a linear correlation between DR and PSA value (Figure 5).

Although DR increased exponentially with PSA value, there was a remarkable increase for PSA beyond 1 ng/ml. For this reason, the consequence of performing an ^18^F-FECH-PET/CT below this value remains questionable.

On the other side, we found an expansion in recurrence spectrum with increasing PSA, so ^18^F-FECH-PET/CT detected 5 lymph node involvements and two lung metastases in 39 patients with PSA less than 1 ng/ml versus no case of local recurrence or bone involvement. However, in 51 patients with PSA less than 1.5 ng/ml, ^18^F-FECH-PET/CT detected 12 lymph node metastases, 2 bone metastases, and 2 lung metastases without any case of local recurrence.

In 96 patients with PSA value less than 2 ng/ml, ^18^F-FECH-PET/CT detected 27 lymph node metastases vs. 3 local recurrences, 2 bone metastases, and 2 lung metastases. In 123 patients with PSA value less than 3 ng/ml, there was evidence of 36 lymph node metastases, 11 local recurrences, 2 lung metastases, and 2 bone metastases.

### 3.3. PSA in Positive Finding vs. Negative Finding

Overall, the median PSA in positive findings was 5.91 (range 0.26–28). By contrast, in negative findings, the median PSA value was 1.6 (range 0.02–9). Statistically, there is a significant difference between both values (*p* < 0.001).

### 3.4. PSA in Different Recurrence Sites

PSA value appears to be different between the different kinds of recurrences. This variation was obvious in comparison between the bone involvement and both kinds of nonbone involvement, namely, LN-R and LR. PSA median in patients with bone involvement was 10.7 (range 1–27.5) vs. 5.1 (range 0.26–28) and 4.4 (range 1.1–14) in patients with lymph node recurrence and local recurrence, respectively. Statistically, there was a significant difference (*p* < 0.05) between bone and nonbone involvement.

On the other hand, both kinds of soft tissue involvements (LN-R and LR) have an analogous PSA value, and no significant difference was demonstrated (*p*=0.41).

### 3.5. Results in relation with Initial Therapy

Initially, the patients underwent different kinds of therapy including radical prostatectomy, radiotherapy, and HIFU. The local recurrence was the most frequent recurrence (>70%) after pure radiation therapy. By contrast, after radical prostatectomy, the lymph node involvement was the most popular with a percentage of >60%, and here, the local recurrence was the second most recurrence. Patients after radical prostatectomy followed by salvage radiotherapy showed a low likelihood of local recurrence. Bone involvement was prominent in patients with a PSA relapse under ongoing AHT.

Likewise, detection rate showed a variation in relation to initial therapy, as demonstrated in Table 1. DR was 46% in patients after radical prostatectomy, 57% after radical prostatectomy followed by radiotherapy, and 69% in patients after radiation alone.

## 4. Discussion

Monitoring the PSA serum level is the main method in the follow-up of PCA patients after initial therapy. In presence of biochemical recurrence, it is of great value to localize the tumor lesions, which have an important role in the early selection of the appropriate therapy, be it either systemic or local.

Detection of recurrence causing a slight PSA relapse remains challenging; at this point, the morphological imaging modalities are of negligible role. The superiority of ^18^F-choline-PET/CT over morphological methods has been demonstrated in a study of Choueiri et al. in 292 patients [13]. They showed that CT, MR, and bone scans are unlikely to be useful when PSA value is lower than 5 ng/ml. By contrast, 66% of the positive results in our study had a PSA value <5 ng/ml.

Independent of the applied procedure, either choline PET/CT or conventional modalities, the PSA level has a determinant role in imaging outcome, which means sensitivity can be enormously altered in the light of existing PSA value. In this setting, many studies had been performed to disclose the role of PSA level in predicting the likelihood of imaging to be positive.

Husarik et al. [15] reported a 71% detection rate in patients with PSA levels ≤2 *μ*g/l, however with a low patient number (*n* = 14). By contrast, we have positive findings in 84 out of 193 patients with an overall detection rate of 44%, similar to the result of Krause et al. using C11-choline [16].

In patients with PSA values less than 1.5 ng/ml, we found a detection rate of 32%, matching the study performed by Castellucci et al. using 11C-choline, who reported a detection rate of 28% in PSA value less than 1.5 ng/ml [17].

The slight PSA increase is supposed to be due to microscopic recurrence; here, FECH-PET did not turn out to be of value and demonstrated a limited capability. For this reason, the consequence of performing the FECH-PET in patients with low PSA level remains questionable. The optimal timing for performing imaging tests after biochemical failure is not well established because the imaging outcome is also affected by many other factors besides the PSA value alone, e.g., PSA doubling time. While Krause et al. [16] reported detection rate of 11C-choline PET/CT of 36% in patients with PSA value <1 ng/ml, Schillaci and his colleagues [18] found a detection rate of 11C-choline PET/CT by 20% in the patient group with PSA value ≤1.1 ng/ml. On the other hand, Castellucci et al. [19] reported a detection rate of 11C-choline PET/CT by 12% with PSA value <1 ng/ml. So, there is a strong variability in the detection rate under <1 ng/ml even by using the same tracer. In this respect, Picchio et al. [20] recommended not to perform choline PET with PSA value less than 1 ng/ml; in our results, there was a remarkable increase in the detection rate with PSA value over 1 ng/ml (Figure 5).

In positive findings, the PSA mean appears to be different in different kinds of recurrences. This difference was obvious in comparison between bone and nonbone involvement. However, in clinical routine, it may not be reasonable to make a guess regarding the recurrence site based on PSA level. So, a low PSA level is neither adequate to rule out bone involvement, nor the high PSA level is sufficient to confirm it, simply because the bone and nonbone involvement can share the same low PSA level.

In another topic, the choline uptake as mentioned earlier is not tumor specific, so it may accumulate in many malignant or benign processes not related with PCA. We described a few false-positive findings due to meningioma, lung cancer, and aneurysm whose presence should be considered in interpreting the PET findings. In this subject, Schillaci et al. found 15 out of 80 patients (18.7%) with findings not suggestive for PCA manifestations [21]. These findings were mainly related to sites of inflammation.

### 4.1. Is the PSA Value the Sole Affecting Factor on Imaging Outcome?

Of course not, because at the same level of PSA, the detection rate of choline PET shows variation based on the site of recurrence. This means whether the examination is supposed to detect local recurrence or other recurrences, choline PET is not equivalent even in the same PSA level.

Although the results show an overall improvement in ^18^F-FECH-PET/CT sensitivity with PSA increase, this improvement seems not to be at the same extent in different types of recurrence.

Indeed, the PSA level is correlated with recurrence volume. The latter should reach a certain value to be detectable, and this value seems to be different between recurrence types, in evidence of the previously described PSA value variation in bone and nonbone involvement. For example, the bone involvement was characterized with a high PSA level, which means to some extent that bone metastases were detected mostly at a higher PSA level and often not detectable in a low level.

In other words, an increase in PSA value to the limit where it can be detectable is not the same between different recurrence sites. So, the probable recurrence site should be considered as a factor that can affect the sensitivity of ^18^F-FECH-PET/CT besides the PSA value. This diagnosis discrepancy was not only demonstrated in bone involvement vs. nonbone involvement but also between both kinds of soft tissue involvement.

Scattoni and coworkers [22] described using 11C-choline PET/CT encouraging results in detecting lymph node recurrence in 25 patients with biochemical relapse (PSA mean: 1.98 ng/ml). They found a 90% detection rate with a high-positive predictive value. By contrast, in detecting the local recurrence, Reske et al. [23] reported in 36 patients (PSA mean: 2.0 ng/ml) a detection rate of 70%.

Certainly, the variance between the abovementioned studies (Scattoni and Reske) can be explained through dissimilarity in study conditions. However, after considering our results, the variance can indicate a real difference in diagnosis capabilities in different clinical manifestations.

### 4.2. Impact of Initial Therapy on Recurrence and Detection Rate

Several therapy options are available for prostate cancer in early stage, varying between radical prostatectomy, percutaneous radiotherapy, or HIFU. Treatment option is mostly selected individually considering the expected side effects.

Since our patients underwent a lot of different therapies, it was of value to identify, whereas such variation has an impact on imaging outcome.

Indeed, patients who underwent radiotherapy alone were more susceptible to develop local recurrence than others. For this reason, the performance of a dynamic study is of a special value and worth to be focused on in patients with a PSA relapse after sole radiotherapy.

On the contrary, patients after radical prostatectomy followed by salvage radiotherapy showed a low-likelihood of local recurrence, which in turn raises the question about the necessity to do a dynamic study in such patients considering the very low-likelihood of local recurrence.

The lymph node recurrence was the most repeated type in case of an operative procedure alone. Likewise, the detection rate differs with the performed procedure. However, it may be attributed to the accompanied variation in PSA value rather to be linked directly with the therapy type.

### 4.3. Limitations Facing the Detection of the Lymph Node Recurrence and Local Recurrence

In 2006, Häcker et al. demonstrated the weakness of ^18^F-FECH-PET/CT in a cohort of 20 patients in searching for occult lymph node metastases [24]. Similar results were published by Scattoni et al. [22]. They reported a low negative predictive value of 11C-choline in detecting lymph node recurrence, and all false-negative results were due to microscopic diseases.

On the other hand, the urinary excretion of the tracer is one of many critical difficulties that have to be kept in mind in looking for a local recurrence in prostate bed or basal bladder wall. This occurs, however, with delay, yielding a chance to take a look at the prostate bed during the first minutes after tracer injection before the entrance of radioactivity into the bladder. This deficiency of ^18^F-FECH-PET/CT can be compensated by adding a dynamic phase at the beginning of the study.

In this topic, it is worth to raise the following question: since TRUS and/or pelvic endorectal MRI remain the first procedures when local recurrence is suspected, is choline PET still of value in this setting?

Vees et al. [25] described the role of F-18-choline and C-11-acetate in 20 patients with suspected residual or recurrent prostate cancer after radical prostatectomy with PSA levels <1 ng/ml. They reported a detection rate of 50% and a superiority of endorectal MRI over choline PET and recommended not to consider it as a standard diagnostic tool. However, it should be pointed out that ^18^F-choline-PET is able to differentiate between radiation-induced injury and local tumor recurrence [26].

### 4.4. Is ^18^F-FECH-PET/CT Still of Value in case of Low Rise in PSA after Prostatectomy?

The American Society for Therapeutic Radiology and Oncology recommend to perform local salvage RT preferentially for patients with PSA <1.5 ng/ml [27]. So, why to perform ^18^F-FECH-PET/CT in patients referred with PSA value less than 1.5 ng/ml even though the distant metastases rate is very low? Castellucci et al. reported a detection rate of 28% (29 out of 102) in their study on patients with a mild PSA increase <1.5 ng/ml [17]. In fact, just 7 of 102 patients (6.8%) are confirmed to have local relapse, and the others have a relapse outside of the prostate bed (13 patients had bone metastases and 9 lymph node metastasis).

At the same PSA level (<1.5 ng/ml), we have similar results. So, the local relapse seems not to be the single reason after prostatectomy causing a slight PSA increasing <1.5 ng/ml. This highlights the importance to perform a whole-body check and not to be satisfied with salvage therapy. Thus, the procedures concerning the status of the prostate bed, e.g., endorectal coil MRT, cannot be a surrogate of a whole-body test even in low PSA. In this case (low PSA value), many factors play a role in the PET efficiency other than the absolute PSA value, such as PSA kinetics and the primary staging.

## 5. Conclusions

PSA value was confirmed to play a determinant role in ^18^F-FECH-PET outcome. However, it was not the sole factor in this topic and others such as the initial therapy should be kept in mind. Moreover, there was a latent link between recurrence type and initial therapy, which if prospectively confirmed may play a guiding role in selecting the appropriate diagnostic methods.

## Figures and Tables

**Table 1:** Detection rate in patients classified based on the initial therapy.

IT	No.	DR (%)	MFR	PSA mean
RP	107	46	LN-R	4.63
RP + RT	35	57	LN-R	7.7
RT	37	69	LR	5.6
HIFU	8	62	LR	4.5
AHT	6	100	OSS	11.01

IT: initial therapy; MFR: most frequent recurrence; RP: radical prostatectomy; RT: radiotherapy; AHT: antihormonal therapy; HIFU: high-intensity focused ultrasound.

## Data Availability

The data used to support the findings of this study are incorporated within the article.
